# Upregulation of* Mitf* by Phenolic Compounds-Rich* Cymbopogon schoenanthus* Treatment Promotes Melanogenesis in B16 Melanoma Cells and Human Epidermal Melanocytes

**DOI:** 10.1155/2017/8303671

**Published:** 2017-11-21

**Authors:** Myra O. Villareal, Sayuri Kume, Mohamed Neffati, Hiroko Isoda

**Affiliations:** ^1^Faculty of Life and Environmental Sciences, University of Tsukuba, Tsukuba City 305-8572, Japan; ^2^Alliance for Research on North Africa (ARENA), University of Tsukuba, Tsukuba City 305-8572, Japan; ^3^School of Life and Environmental Sciences, University of Tsukuba, Tsukuba City 305-8572, Japan; ^4^The Institute of Arid Region (IRA), Medenine, Tunisia

## Abstract

Melanin provides inherent protection against skin cancer by absorbing broad-spectrum radiant energy of UV radiation. Cutaneous malignant melanoma incidence has recently been observed to increase and the frequency is closely associated with the skin color, highlighting the importance of skin pigmentation. Here, we showed how melanin biosynthesis is enhanced by treatment with phenolic compounds-rich* Cymbopogon schoenanthus* (CYM) in B16 murine melanoma cells and human epidermal melanocytes (HEM). CYM increased the melanin content of the cells by upregulating the expression of tyrosinase (TYR), tyrosinase-related protein 1 (TRP1), and dopachrome tautomerase (DCT) at the protein and mRNA levels, comparable to the effect of *α*-melanocyte-stimulating hormone (MSH), in both B16 cells and HEM. Moreover, global gene expression analysis showed that at least 44 pigmentation-associated genes were modulated, including the microphthalmia-associated transcription factor* (Mitf)* and its transcriptional regulators (*Sox10*,* Pax3*, and* Lef1*). Upregulation of copper transport-associated gene* Atp7b* indicates that CYM also promotes tyrosinase activity. CYM upregulated* Mitf* and possibly activates tyrosinase enzyme, providing evidence for its possible use to promote melanogenesis and as a therapeutic agent against hypopigmentation disorders.

## 1. Introduction

Skin pigmentation is our first line of defense against the harmful UV radiation, which is considered as one of the major risk factors for melanoma skin cancer. Annually, 132,000 individuals are diagnosed with melanoma, and it is believed that as the ozone layers are depleted, more UV radiation from the sun reaches the earth and will increase the incidence of skin cancers [1]. Moreover, the increase in sunburn and the use of tanning beds have been reported to be in an alarming situation now and contribute to the increase in the risk of cutaneous melanoma, especially in young individuals and in predominately fair-skinned populations [2, 3]. The amount of melanin produced by melanocytes is considered to be the most useful predictor of risk for skin cancer so that highly pigmented skin provides protection against skin cancer by 500–1000-fold [4, 5]. Melanocytes, which contain specialized organelles called melanosomes, produce the pigment melanin. Melanins are strong light scatterers with a broad UV-visible absorption spectrum, providing protection not just to melanocytes but also to keratinocytes and other cells in the epidermis against UV radiation-induced damage (photoproducts and oxidative stress). Melanin can absorb a portion of the UV energy and transform it into heat that is dissipated into the body [6, 7]. Melanin biosynthesis occurs through an oxidation process, starting with the amino acid L-tyrosine, with the rate-limiting enzyme tyrosinase (TYR), catalyzing three distinct reactions: hydroxylation of L-tyrosine, dehydrogenation of L-DOPA, and dehydrogenation of DHI, with L-DOPA as the cofactor in the first and third reactions [4, 8, 9]. Other reactions are mediated by tyrosinase-related protein (TRP1) and dopachrome tautomerase (DCT) that catalyze the tautomerization of indolene-2-carboxylic acid-5, 6-quinone or dopachrome and oxidation of 5,6-dihydroxylindole-2-carboxylic acid, respectively [10]. The expression of the melanogenic enzymes* Tyr*,* Trp1*, and* Dct* is regulated by the helix-loop-helix transcription factor microphthalmia-associated transcription factor* (Mitf)*.

Recent researches focus on the regulation of pigmentation at the molecular level to promote melanogenesis as a possible therapeutic strategy against UV-induced DNA damage and skin cancer. Since individuals with lighter skin color are more likely to develop skin cancer than those with darker skin color [11], increasing skin pigmentation using materials considered to be safe for use can therefore be considered as the best strategy to provide protection against UV or to attain darker skin color without resorting to use of skin cancer-inducing tanning beds.

Recently, the acceptance into mainstream medicine of mixtures of combination of plant natural products that may affect multiple pharmacological targets beyond the reach of single-compound-based drugs has been suggested [12]. We have previously reported that ethanol extract of* Cymbopogon schoenanthus* (CYM) provides beneficial effect against stress in ICR mice subjected to forced swimming and tail suspension tests and the effect was attributed to its major components quercetin-3-rhamnoside, trans-cinnamic acid, resorcinol, caffeic acid, 2.5-dihydroxybenzoic acid, ferulic acid, and gallic acid [13]. In pigment cells, oxidative stress causes a downregulation of the melanogenic enzymes TYR, TRP1, and DCT and their transcription factor MITF, leading to decreased melanin biosynthesis [14, 15]. And although the anti-stress effect of CYM has been tested, its effect on melanogenesis has not yet been determined. Therefore it is of major interest to characterize how CYM can regulate pigment cell function and identify the molecular mechanism underlying its effect. In this study, the effect of CYM on melanogenesis in B16 cells and human epidermal melanocytes was evaluated.

## 2. Materials and Methods

### 2.1. Cell Lines and Cell Culture

Murine B16F10 melanoma cells or B16 cells (Cell No. RCB2630) used in this study were obtained from the Riken Cell Bank (Tsukuba, Japan) and maintained in Dulbecco's modified Eagle's medium (Nissui, Tokyo, Japan) supplemented with 10% fetal bovine serum (Sigma, St. Louis, MO, USA), 4 mM L-glutamine (Sigma), 50 units/mL penicillin, and 50 *μ*g/mL streptomycin (Cambrex, East Rutherford, NJ, USA) and incubated at 37°C in a humidified atmosphere of 5% CO_2_. Human epidermal melanocytes (HEM) were obtained from Cell Applications, Inc. (San Diego, CA, USA) (Cat. No. 104K-05a), maintained in melanocyte growth medium (Cell Applications, Inc.), incubated at 37°C in a 5% CO_2_ humidified incubator, and expanded for at least five passages. The medium was changed twice a week and subcultured when it was about 80% confluent.

### 2.2. Plant Samples, Preparation, and Extraction

Leaves of* Cymbopogon schoenanthus* sp. were collected from Tunisia and identified at the Ecology Laboratory, Institut des Regions Arides (IRA), Tunisia, while voucher specimens of the leaf samples (UT-ARENA-00709) were deposited in the Alliance for Research on North Africa, University of Tsukuba, Japan. Extract of* C. schoenanthus* (CYM) was prepared as previously reported [13]. Briefly, 10 g of leaves of air-dried C.* schoenanthus* was macerated in 100 ml 70% ethanol and kept for two weeks at room temperature. The liquid fraction was then collected and, prior to use, was filtered using a 0.22 *μ*m filter (Millipore, USA) and stored at −80°C. As a reference or positive control, 400 nm *α*-MSH or [Nle^4^, D-Phe^7^]-D-Phel, melanocyte-stimulating hormone trifluoroacetate salt (≥95% purity) purchased from Sigma was used.

### 2.3. Cell Proliferation Assay

The effect of CYM on B16 cells proliferation was assessed using the 3-(4,5-dimethylthiazol-2-yl)-2,5-diphenyl tetrazolium bromide or MTT assay (Dojindo, Japan). B16 cells (3 × 10^4^ cells/well) were seeded onto 96-well plates and cultured as described above. After 24 h of incubation, the medium was replaced with a fresh medium with or without the sample at various concentrations. MTT (5 mg/ml) was then added; the plates covered with aluminum foil and were incubated further for 28 h. Sodium dodecyl sulphate (SDS; 10%) was then added at 100 *μ*l per well and incubated overnight at 37°C to dissolve the formazan completely. Absorbances were obtained at 570 nm using a microplate reader (Powerscan HT; Dainippon Pharmaceuticals USA Corp., NJ, USA). To correct the absorbances, blanks containing only medium MTT and SDS were used.

### 2.4. Melanin Assay

B16 cells were cultured at a density of 5 × 10^5^ cells per 100-mm Petri dish. After overnight incubation, the culture medium was replaced with DMEM medium containing either 400 nM *α*-MSH or different dilutions of* C. schoenanthus* ethanol extract. Cells were treated with *α*-MSH served as positive control. After incubation for 48 h, the medium was then removed and the cells were washed twice with phosphate-buffered saline (PBS) and harvested by trypsinization (0.25% trypsin/0.02% EDTA in PBS; Sigma). The harvested cells were pelleted and solubilized using 0.1% Triton X-100 and then purified by precipitation in 10% trichloroacetic acid. The isolated melanin was then dissolved in 8 m NaOH, followed by incubation for 2 h at 80°C. The amount of melanin in the solution was determined spectrophotometrically at 410 nm. The total melanin content was estimated using the standard curve for synthetic melanin and expressed on a per viable cell basis and as percentage of control. The cell viability and total number of cells were assessed using the Guava PCA ViaCount Program (GE Healthcare, UK, Ltd., Buckinghamshire, UK).

### 2.5. Western Blotting

B16 cells or HEM were treated with or without CYM or *α*-MSH for 24 h and the protein samples were extracted using radio-immunoprecipitation assay (RIPA) buffer (Sigma, USA) following the manufacturer's instructions. Protein samples (10 *μ*g) were resolved in 10% sodium dodecyl sulphate-polyacrylamide gel by electrophoresis (SDS-PAGE), transferred to PVDF membrane (Merck Millipore, USA), and blotted with primary antibodies for tyrosinase (TYR), tyrosinase-related protein 1 (TRP1), dopachrome tautomerase (DCT), and GAPDH. All the antibodies were obtained from Santa Cruz Biotechnology, Inc., USA with catalog numbers C-19 (TYR), G-9 (TRP1), DCT (B-7), and SC-32233 (GAPDH). The signal was visualized using LI-COR Odyssey Infrared Imaging System after reaction with goat anti-mouse IRDye 680LT, donkey anti-goat IRDye 800CW (LI-COR), or goat anti-rabbit IRDye 800CW (LI-COR). The band intensities in western blots were determined using LI-COR Odyssey System.

### 2.6. DNA Microarray

To determine the mechanism underlying the effect of* C. schoenanthus* on melanogenesis, a genome-wide expression analysis was performed using Affymetrix (Santa Clara, CA, USA) GeneChip Mouse Genome 430 2.0 Array following the manufacturer's instructions. Labeled B16 cRNAs (1 *μ*g) from CYM-treated and control cells were amplified, labeled, and hybridized onto the array. Analysis of gene significance was done using Partek® Express™ Software (Partek, Inc., Chesterfield, MO, USA) while Mouse Genome Informatics (MGI) was used as a database resource for the GO annotations. Changes in gene expression were considered significant when up- or downregulated by at least 1.5-fold by CYM versus control (untreated). Pathway Studio analysis was done using Elsevier Pathway Studio 11.2.5.6.

### 2.7. Quantitative Real-Time PCR

Total RNA was extracted using Isogen reagent (Nippon Gene, Tokyo, Japan) following the manufacturer's instructions. RNA (1 *μ*g) was reverse-transcribed using the SuperScript III reverse transcriptase kit (Invitrogen, Carlsbad, CA, USA). Primers specific to tyrosinase* (Tyr)*, tyrosinase-related protein 1* (Trp1)*, dopachrome tautomerase* (Dct)*, and microphthalmia-associated transcription factor* (Mitf)* were used for quantitative real-time PCR performed with a 7500 Fast Real-time PCR system using TaqMan Universal PCR mix and TaqMan probes (Applied Biosystems, Foster City, CA, USA). All reactions were run in triplicate, and data were analyzed using the 2^−ΔΔCT^ values method.

### 2.8. Statistical Evaluation

Mean values ± SEM were calculated from three samples of at least three trials. The statistical analyses of the results were performed using Student's *t*-test to determine the significance of results of the treated cells versus that of the control (untreated). A value of *P* ≤ 0.05 was considered significant.

## 3. Results

### 3.1. CYM Stimulates Melanin Biosynthesis in B16 Cells

**Figure 1:**
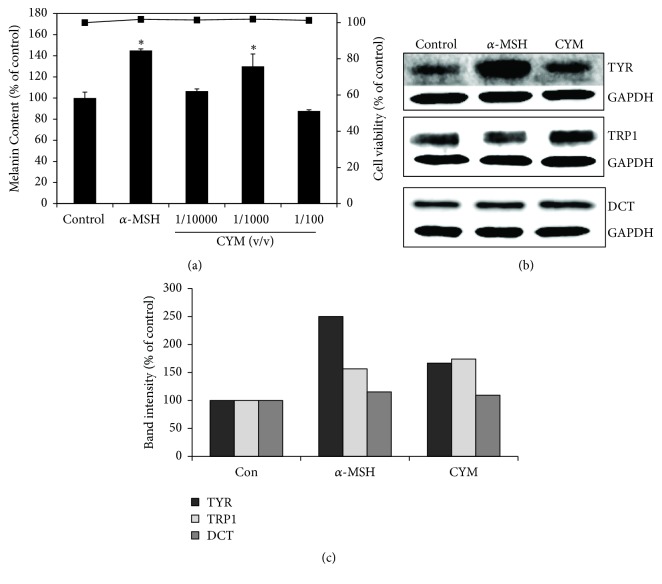
Effect of* Cymbopogon schoenanthus* ethanol extract on melanogenesis in B16 cells. (a) Melanin content (bar graph) and the cell viability (line graph) of B16 cells treated without (control) or with* C. schoenanthus* ethanol extract (CYM) at various concentrations (1/10000, 1/1000, or 1/100 v/v). (b) The effect of CYM on the expression of the tyrosinase (TYR), tyrosinase-related protein 1 (TRP1), and dopachrome tautomerase (DCT). The signals were visualized using LI-COR Odyssey Infrared Imaging System after reaction with goat anti-mouse IRDye 680LT or goat anti-rabbit IRDye 800CW (LI-COR). Results represent the mean ± SD of triplicate samples. ^*∗*^Statistically significant (*P* ≤ 0.05) difference between treated cells and control. (c) Protein quantification based on the band intensities from the western blot in (b) determined using the LI-COR system.

The melanin assay results showed an increase in the melanin content of B16 cells treated with CYM. CYM at 1/10,000 v/v had higher melanin content compared to the control (by 6%) while 1/1000 v/v CYM increased the melanin content by 30%, 48 h after treatment (Figure 1(a)). CYM at 1/100 v/v appeared to have lower melanin content but this was due to the slight cytotoxicity of the extract at this concentration. The viability of the cells used in the quantification of intracellular melanin was not affected by 1/10,000 v/v and 1/1,000 v/v CYM. For the succeeding experiments, CYM at 1/1,000 v/v was used.

### 3.2. CYM Increases the Expression of Melanogenic Enzymes in B16 Cells

To determine if the increase in melanogenesis was caused by an increase in the melanogenic enzymes tyrosinase (TYR), tyrosinase-related protein 1 (TRP1), or dopachrome tautomerase (DCT) expression, total protein of B16 cells treated with or without 1/1,000 v/v CYM or *α*-MSH was extracted and western blotting was carried out. Results showed that, compared with the control, CYM promoted the expression of TYR, TRP1, and DCT, although the increase is more pronounced in TYR and TRP1 than DCT. The expression levels of all the melanogenic enzymes were increased by *α*-MSH (Figures 1(b) and 1(c)).

### 3.3. CYM Promoted Melanin Biosynthesis in Human Epidermal Melanocytes

**Figure 2:**
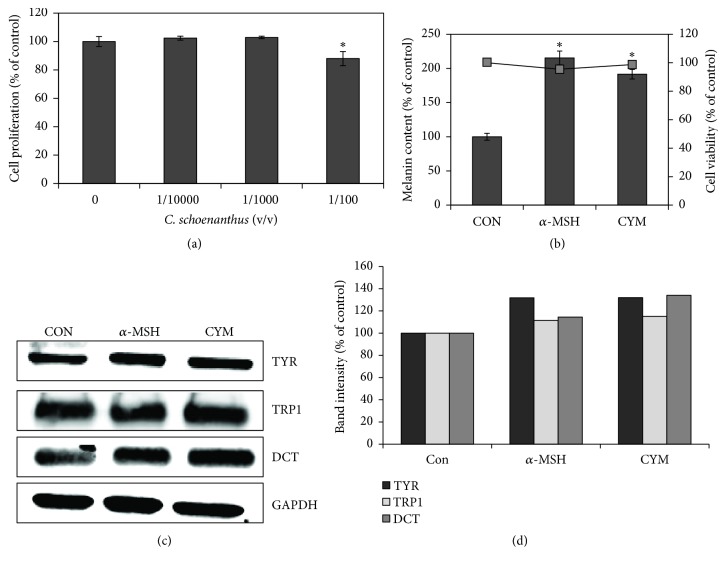
Effect of* Cymbopogon schoenanthus* ethanol extract on melanogenesis in human epidermal melanocytes (HEM). (a) Cell proliferation evaluated using MTT Assay: HEM (3 × 10^3^ cells/well) were treated with* C. schoenanthus* ethanol extract (CYM) at various concentrations (0, 1/10000, 1/1000, or 1/100 v/v) and incubated for 48 h. (b) Melanin content was determined by seeding HEM in 100 mm dish (5 × 10^5^ cells/dish) and treated without (control) or with 1/1000 (v/v) CYM and incubated for 72 h. The bar graph indicates the melanin content (left-hand *y*-axis) while the line graph indicates cell viability (right-hand *y*-axis). (c) Expression of the tyrosinase (TYR), tyrosinase-related protein 1 (TRP1), and dopachrome tautomerase (DCT) was determined using western blotting. Total protein was extracted from HEM cultured in 100 mm Petri dish (3 × 10^6^ cells/dish) and treated without (Con) or with 400 nm alpha-melanocyte-stimulating hormone (*α*-MSH) or 1/1000 (v/v) CYM for 48 h. Results represent the mean ± SD of triplicate determinations. ^*∗*^Statistically significant (*P* ≤ 0.05) difference between control and treated cells. (d) Protein quantification based on the band intensities from the western blot in (c) determined using the LI-COR system.

To determine if the observed effect of CYM on melanin biosynthesis on murine B16 cells is the same on human pigment cell, the melanin content of human epidermal melanocytes (HEM) treated without or with CYM was determined. Results of MTT assay, used to determine noncytotoxic concentrations of HEM, showed that CYM has no cytotoxic effect on HEM at 1/10,000 and 1/1,000 v/v while 1/100 v/v has slight cytotoxicity and decreased melanocyte proliferation by about 10% (Figure 2(a)). CYM at 1/1000 v/v was then used in the succeeding experiments. Melanin assay results showed that CYM treatment induced a twofold increase in the melanin content of HEM (bar graph) without affecting the cell viability (line graph) (Figure 2(b)). However, unlike in the B16 cells, the growth medium color of CYM-treated cells was observed to be darker in color compared to the untreated cells' growth medium prompting us to quantify the intracellular melanin and the melanin released into the growth medium. Like in B16 cells, the expression of TYR, TRP1, and DCT was also increased by CYM. The expression of TYR and DCT was greater than that of TRP1 in both CYM- and *α*-MSH-treated HEM (Figures 2(c) and 2(d)).

### 3.4. Transcriptome Changes Induced by CYM

The gene expression profile of B16 cells treated with CYM relative to control (untreated cells) was determined using microarrays that contained 39,000 transcripts targeting mouse genes. CYM modulated a total of 11,746 genes. Gene ontology (GO) and function (biological process) of the modulated genes were identified using Mouse Genome Informatics (MGI) and the results showed that CYM differentially altered the expression of genes associated with pigmentation, cytoskeleton organization, positive JUN kinase activity, and neuron differentiation. Moreover, genes relevant in the metabolic process, protein transport, negative regulation of cAMP-dependent protein kinase activity, and cell adhesion were downregulated. A summary of the pigmentation-associated genes modulated by CYM treatment. (Table 1) showed that CYM upregulated the expression of the three melanogenic enzymes* Tyr*,* Trp1*, and* Dct* as well as cytoskeleton organization-associated genes, copper transport and most melanosome transport genes such as thymosin beta 10* (TMSB 10)* and ribosomal protein S3* (Rps3)*. The microphthalmia-associated transcription factor* (Mitf)* was upregulated 2-fold. As expected, most of the genes regulated by* Mitf* were also upregulated* (Lamp1*,* Tyr*,* Trp1*,* Dct*,* Mlph*,* Mc1r*,* Mlana*,* Slc45a*,* Rab27a*,* Tnfrsf14, Irf4, Lamp3, *and* Shc4)* although some were downregulated* (Bcl2*,* Ednrb*,* Tbx2,* and* Hk2)*.* Mlana*,* Mlph*,* Pmel*, and* Rab38* are significant for melanosome component or transport and copper ion transport. Genes significant for homeostasis were also upregulated* (Atox1*,* Atp7b)*. Upregulation of the transcription factors* Pax3*,* Sox10*, and* Lef1* is the most likely cause for the observed increase in* Mitf *expression. Elsevier Pathway Studio analysis further revealed that CYM also affected the following signal transduction pathways: FOX1, mTOR, insulin, JNK/MAPK, and Hippo/YAP1 (data not shown).

### 3.5. Validation of the Microarray Results

**Figure 3:**
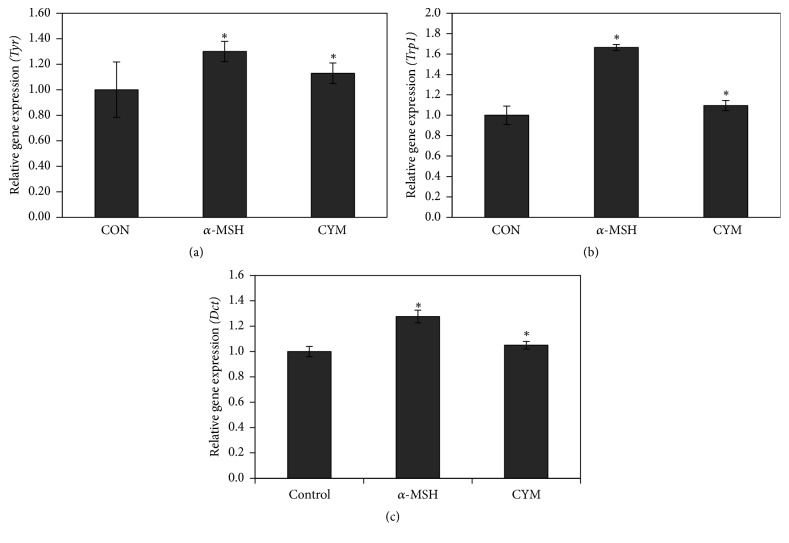
Effect of* Cymbopogon schoenanthus* ethanol extract on the mRNA expression level of melanogenic enzymes: (a) tyrosinase* (Tyr)*, (b) tyrosinase-related protein 1* (Trp1)*, and (c) dopachrome tautomerase* (Dct)* determined using TaqMan real-time quantitative PCR. B16 cells were cultured in 100 mm dish (3 × 10^6^ cells/dish) and treated without (CON) or with 1/1000 (v/v)* C. schoenanthus* ethanol extract (CYM), using 400 nm alpha-melanocyte-stimulating hormone (*α*-MSH) as a positive control and incubated for 4 h after which RNA was extracted, and then reverse transcription PCR was carried out to obtain cDNAs that were used for real-time PCR (ABI 7500 Fast Real-time PCR system). Results represent the mean ± SD of three independent experiments. ^*∗*^Statistically significant (*P* ≤ 0.05) difference between control and treated cells.

**Figure 4:**
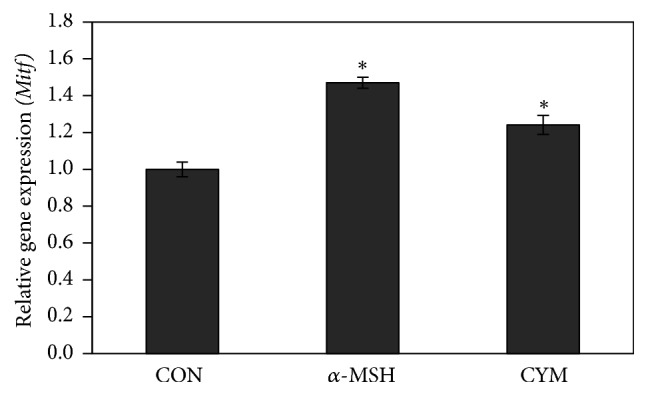
Effect of* Cymbopogon schoenanthus* ethanol extract on the mRNA expression level of microphthalmia-associated transcription factor* (Mitf)* determined using TaqMan real-time quantitative PCR. B16 cells were cultured in 100 mm dish (3 × 10^6^ cells/dish) and treated without (CON) or with 1/1000 (v/v)* C. schoenanthus* ethanol extract (CYM), using 400 nm alpha-melanocyte-stimulating hormone (*α*-MSH) as a positive control, and incubated for 4 h after which RNA was extracted, and then reverse transcription PCR was carried out to obtain cDNAs that were used for real-time PCR (ABI 7500 Fast Real-time PCR system). Results represent the mean ± SD of three independent experiments. ^*∗*^Statistically significant (*P* ≤ 0.05) difference between control and treated cells.

Quantifying the mRNA expression level of the melanogenic enzymes using real-time PCR validated the microarray results. Results showed that the mRNA levels of* Tyr* and* Trp1* genes were significantly increased, 4 h after treatment with CYM or *α*-MSH compared to the control (Figures 3(a) and 3(b)) while* Dct* gene expression was only slightly increased (5% increase compared to the control) (Figure 3(c)). Real-time PCR results showed that* Mitf* mRNA expression was increased 4 h after treatment with CYM and *α*-MSH (Figure 4).

## 4. Discussion

Mammalian skin pigmentation is a result of the biosynthesis and accumulation of epidermal melanin. Melanin is the most important photoprotective factor due to its ability to absorb UV radiation (UVR) and has antioxidant and reactive oxygen species (ROS) scavenging properties [16]. The production of melanin, therefore, is critical for inhibiting melanoma, as exposure to UVR, especially the UVA component, and is known to initiate oxidative stress in human skin [17, 18]. And even though there are cosmetics or products, that claim to provide sun protection, there is no better protection that that imparted by melanin itself. Recently, there is an increase in the number of researches devoted to the evaluation of natural products or plant extracts to regulate melanogenesis [19, 20].

Here we demonstrated the melanogenesis regulatory effect of* Cymbopogon schoenanthus* (CYM) ethanol extract on B16 cells (Figures 1(a) and 1(b)). The enhanced melanogenesis observed in B16 cells was also seen in human epidermal melanocytes (HEM) treated with CYM (Figure 2(b)). Differences between the promoter regions of mouse and human tyrosinase have been reported [21, 22], but CYM appears to promote the production of both murine and human tyrosinase without cytotoxicity (Figures 2(a), 2(b), and 2(c)). Melanogenesis is the unique process of producing the melanin within melanosomes and is a major function of both differentiated normal melanocytes and malignant melanoma cells [23]. There are two kinds of melanins, the eumelanin (black-to-brown) and the pheomelanin (yellow-to-reddish-brown). Melanins are derived from dopaquinone (DQ). When L-tyrosine is oxidized by TYR, it forms an intermediate L-3, 4-dihydroxyphenylalanine (DOPA). Cyclization of DQ's quinone produce cyclodopa which, following another redox exchange with another molecule of DQ, produces DOPAchrome and DOPA, and decomposition of DOPAchrome that produces 5,6-dihydroxyindole and 5,6-dihydroxyindole-2-carboxylic acid (DHICA). DHI and DHICA undergo further oxidation and polymerization to form eumelanins. DCT catalyzes the tautomerization of DOPAchrome to produce DHICA. TYR mediates the oxidation of DHICA to the quinone form in humans and by TRP1 in mice [24]. In this study, *α*-melanocyte-stimulating hormone (*α*-MSH) was used as a positive control owing to its ability to induce melanocyte differentiation and melanogenesis [25].

When melanocytes are exposed to oxidative stress, however, melanogenesis is inhibited because the melanogenic enzymes or melanocyte differentiation markers TYR, TRP1, and DCT and the melanogenesis master regulator MITF [15] are downregulated. CYM is known for its antioxidant effect owing to its high polyphenol content [26]. Previously we have reported that CYM can alleviate H_2_O_2_-induced cytotoxicity in neurotypic cells, and the effect was attributed to the seven phenolic compounds quercetin-3-rhamnoside, trans-cinnamic acid, resorcinol, caffeic acid, and 2.5-dihydroxybenzoic acid, ferulic acid, and gallic acid present in the extract [13]. Whether the increase in melanogenesis was a result of the alleviation of oxidative stress in the cells or not is not discussed here but is a possibility. It is clear, however, that the stimulation of melanogenesis was the cause of the synergistic interaction of the bioactive compounds present in CYM.

The regulatory control of melanin biosynthesis is complex and involves different signaling pathways in response to hormones, growth factors, cytokines, and neurotransmitters [27]. CYM modulated the expression of pigmentation-associated genes as well as those that promote cell homeostasis (Table 1). The increase in the expression of the melanogenic enzymes* Tyr*,* Trp1*, and* Dct* expression can be attributed to the increase in the* Mitf* expression, which was increased 2.2-fold.* Mitf* can be activated by several transcription factors such as* Sox10*,* Lef1, and Pax3*, [23] which were all upregulated by CYM by 2.5-, 1.5-, and 1.2-fold, respectively. Another possible reason for the increase in* Mitf* expression was the downregulation of* Map2k1* that is the gene that codes for MAPKs ERK1 and ERK2. The regulation of melanocyte or melanoma or cell differentiation involves the MAP kinase pathway and the activation of ERKs is a required event in the initiation of melanogenesis [28]. Phosphorylation of ERK1/2 promotes reducing melanogenesis by downregulating* Mitf* [25]. Another possible reason for the increased melanogenesis could be the increase in* Atp7b* and* Atox1* genes expression, with a 2.0-fold and 4.8-fold increase, respectively (Table 1).* Atp7b* and* Atox1* genes play an important role in intracellular copper ion transport and cellular copper ion homeostasis. The key melanogenic enzyme tyrosinase is a copper-containing enzyme, with two copper atoms in its active sites, Cu (II) and Cu (I) [29], and has six copper-binding domains in its N-terminal region and believed to accept copper from cytoplasmic carriers and/or assist in delivering copper to the channel [30]. The structure of the key regulatory enzyme of melanogenesis, tyrosinase, shows high homology with other tyrosinase-related proteins including TRP1 and DCT, sharing about 40% amino acid homology with tyrosinase, with which they also have structural similarity [31]. All three melanogenic enzymes are transcriptional targets of MITF, the master regulator of melanocyte development, function, and survival, which binds to elements shared between their respective promoters [23]. The fundamental role of MITF in the regulation of mammalian pigmentation is evidenced by the genetically determined pigmentary disorders resulting from mutation at the Mitf* (Mi)* locus in mice [23, 32]. CYM also differentially downregulated genes associated with signaling pathways that activate* Mitf* (e.g., cAMP pathway), as well as those that bind the ATP and RNA (Table 2).

Components analysis of CYM, using RP-HPLC coupled with UV-Vis multiwave length detector, identified five phenolic compounds quercetin-3-rhamnoside, trans-cinnamic acid, resorcinol, caffeic acid, and 2.5-dihydroxybenzoic acid, ferulic acid, and gallic acid [13]. It is interesting to note that although the components of CYM, individually, have been reported to inhibit melanogenesis [33–35], CYM extract has the opposite effect and suggests that this effect on melanogenesis is due to the synergistic effects between these polyphenols [35, 36]. The validation of the microarray results was done using real-time PCR, the results of which showed that CYM promoted the melanogenic enzymes' gene expression, 4 h after treatment. Increasing the treatment time would most likely cause a significant increase in the expression. CYM has been reported to mitigate oxidative stress but whether the observed increase in melanogenesis was a direct effect of this effect was not determined in this study. A follow-up study will be conducted to see if the melanogenesis promotion effect of CYM was a direct effect of mitigation of oxidative stress.

Plants are valuable source of therapeutic compounds and their extracts' complex composition consists of related compounds with multiple bioactivities, with their synergistic interaction providing greater total bioactivity [37]. There is a recognition now of the therapeutic potential of plant extracts which is essentially a mixture of interacting compounds. In fact medicinal plants are also subjected to clinical tests to evaluate for safety and efficacy in the same way conventional drugs are evaluated, which can be shortened considering their history of safe human use [38].

## 5. Conclusion

CYM can promote melanogenesis in B16 cells and HEM without cytotoxicity. The results of this study suggest that any CYM-containing cosmetic product or intake of CYM would have a pigmentation enhancing effect that may provide protection against harmful UV radiation and, thus, may help prevent melanoma skin cancer. Furthermore, the results in this study suggest that the combination of compounds at the concentration which are present in the extract could provide an insight into the positive interaction between natural compounds present in CYM. Exploring the possible relationship between its antioxidant effect and the melanogenesis-promoting effect of CYM, which is not determined here, would be interesting to explore in future studies. CYM, with its cell differentiation-induction effect on B16 cells and melanocytes, has potential application in developing effective therapeutics against hypopigmentation-related disorders.

## Figures and Tables

**Table 1:** Top ten (10) genes differentially upregulated in B16 cells following treatment with *Cymbopogon schoenanthus* (CYM) ethanol extract (control versus CYM).

Gene symbol	Gene name	Molecular function	Biological Function	Fold change
*Tmsb10*	Thymosin, beta 10	Actin monomer binding	Actin cytoskeleton organization	21.6

*Rplp0*	Ribosomal protein, large, P0	Poly(A) RNA binding	Cellular response to interleukin-4/ribosome biogenesis	20.8

*Tyrp1*	Tyrosinase-related protein 1	Copper ion binding; oxidoreductase activity; protein binding	Melanin metabolic process; melanocyte differentiation; melanosome organization; pigmentation' positive regulation of melanin biosynthetic process	20.2

*Rps3*	Ribosomal protein S3	DNA binding; HSP70 protein binding; HSP90 protein binding; kinase/lyase binding; microtubule binding; NF-kB binding; SUMO binding; nucleic acid binding;	Negative regulation of translation; positive regulation of gene expression; positive regulation of JUN kinase activity; positive regulation f microtubule polymerization; response to oxidative stress; response to TNF agonist; spindle assembly;	19.8

*Ppia*	Predicted pseudogene 9234/peptidylprolyl isomerase A	Cyclosporine A binding; isomerase activity; poly(A) RNA binding	Neuron differentiation (part of oligodendrocyte); lipid particle organization; protein folding;	19.8

*Rplp2*	Ribosomal protein, large P2	Structural constituent of ribosomes;	Translational elongation	19.4

*Vim*	Vimentin	Double-strand RNA binding; glycoprotein binding; kinase binding; protein binding; protein kinase binding	Astrocyte development; Bergmann glial cell differentiation; intermediate filament organization; negative regulation f neuron projection development; positive regulation of glial cell proliferation; regulation of axonogenesis; regulation of Schwann cell migration; SMAD protein signal transduction;	19.0

*Chchd2/Gm13202*	Coiled-coil-helix-coiled-coil-helix domain containing 2/predicted gene 13202	Sequence-specific DNA binding; transcription factor binding;	Positive regulation of transcription from RNA polymerase II promoter; regulation of cellular response to hypoxia; transcription, DNA-templated	19.0

*Dct*	Dopachrome tautomerase	Dopachrome isomerase activity; metal ion binding; oxidoreductase activity	Cell development; developmental pigmentation; melanin biosynthetic process; pigmentation; positive regulation of neuroblast proliferation; ventricular zone neuroblast division; melanin biosynthetic process from tyrosine; metabolic process	19.0

*Actb*	Actin, beta	ATP binding; identical protein binding; kinesin binding;	Axonogenesis; cellular response to electrical stimulus (part of oligodendrocyte, myelin sheath)	18.8

**Table 2:** Top ten (10) genes differentially downregulated in B16 cells following treatment with *Cymbopogon schoenanthus* (CYM) ethanol extract (control versus CYM).

Gene symbol	Gene name	Molecular function	Biological function	Fold change
*Nadk2*	NAD kinase 2, mitochondrial	ATP Binding	Metabolic process; NAD/NADP metabolic process; phosphorylation	−5.5

*Fam21*	Family with sequence similarity 21	Phosphatidylinositol-3,4,5-trisphosphate binding	Protein transport	−5.0

*Rimkla*	Ribosomal modification protein rimK-like family member A	ATP binding; Catalytic activity	Cellular protein modification process	−4.8

*Pkig*	Protein kinase inhibitor, gamma	cAMP-dependent protein kinase inhibitor activity	Negative regulation of cAMP-dependent protein kinase activity; signal transduction	−4.7

*Zg16*	Zymogen granule protein 16	Carbohydrate binding	Protein transport	−4.6

*Zfp60*	Zinc finger protein 60	DNA binding; metal ion binding; nucleic acid binding; transcription factor activity, sequence-specific DNA binding	Regulation of transcription, DNA-templated	−4.5

*Col12a1*	Collagen, type XII, alpha 1		Cell adhesion	−4.4

*Ccdc171*	coiled-coil domain containing 171		Sequence-specific DNA binding transcription factor activity	−4.2

*Nanos1*	Nanos homolog 1 (drosophila)	Zinc ion binding; RNA binding	Positive regulation of nuclear-transcribed mRNA catabolic process, deadenylation-dependent decay; epithelial cell migration	−4.0

*Crkl*	v-crk sarcoma virus CT10 oncogene homolog-like (avian)	RNA binding	Cell differentiation, cellular component organization, cell proliferation	−4.0
